# Region Specific Differences of Claudin-5 Expression in Pediatric Intracranial Ependymomas: Potential Prognostic Role in Supratentorial Cases

**DOI:** 10.1007/s12253-016-0084-3

**Published:** 2016-07-09

**Authors:** József Virág, Christine Haberler, Gábor Baksa, Violetta Piurkó, Zita Hegedüs, Lilla Reiniger, Katalin Bálint, Monika Chocholous, András Kiss, Gábor Lotz, Tibor Glasz, Zsuzsa Schaff, Miklós Garami, Balázs Hegedűs

**Affiliations:** 1 2nd Department of Pediatrics, Semmelweis University, Budapest, Hungary; 2 Institute of Neuropathology, Medical University of Vienna, Vienna, Austria; 3 1st Department of Anatomy, Semmelweis University, Budapest, Hungary; 4 2nd Department of Pathology, Semmelweis University, Üllői út 93, Budapest, H-1091 Hungary; 5 1st Department of Pathology and Experimental Cancer Research, Semmelweis University, Budapest, Hungary; 6 MTA-SE NAP, Brain Metastasis Research Group, Hungarian Academy of Sciences - Semmelweis University, Budapest, Hungary; 7 Department of Pediatrics, Medical University of Vienna, Vienna, Austria; 8 Department of Thoracic Surgery, Medical University of Vienna, Vienna, Austria; 9 Molecular Oncology Research Group, Hungarian Academy of Sciences - Semmelweis University, Budapest, Hungary

**Keywords:** Ependymoma, Claudin-5, Tight junction, Supratentorial, Choroid plexus epithelium, Prognosis

## Abstract

Ependymomas are common pediatric brain tumors that originate from the ependyma and characterized by poor prognosis due to frequent recurrence. However, the current WHO grading system fails to accurately predict outcome. In a retrospective study, we analyzed 54 intracranial pediatric ependymomas and found a significantly higher overall survival in supratentorial cases when compared to infratentorial tumors. Next we performed region-specific immunohistochemical analysis of the ependyma in neonatal and adult ependyma from the central canal of spinal cord to the choroid plexus of lateral ventricles for components of cell-cell junctions including cadherins, claudins and occludin. We found robust claudin-5 expression in the choroid plexus epithelia but not in other compartments of the ependyma. Ultrastructural studies demonstrated distinct regional differences in cell-cell junction organization. Surprisingly, we found that 9 out of 20 supratentorial but not infratentorial ependymomas expressed high levels of the brain endothelial tight junction component claudin-5 in tumor cells. Importantly, we observed an increased overall survival in claudin-5 expressing supratentorial ependymoma. Our data indicates that claudin-5 expressing ependymomas may follow a distinct course of disease. The assessment of claudin-5 expression in ependymoma has the potential to become a useful prognostic marker in this pediatric malignancy.

## Introduction

Ependymomas are the third most common central nervous system (CNS) malignancies in children [1]. The majority of the cases is located in the posterior fossa [2, 3], however in the first years of life they often appear supratentorial [4]. According to the WHO CNS tumor classification ependymal tumors are grouped into grade I (subependymoma, myxopapillary ependymoma), II (ependymoma) and III (anaplastic ependymoma) [5] Whereas patients with subependymoma and myxopapillary ependymoma have in general a very good prognosis, grade II and grade III ependymomas have a relatively poor outcome [6]. The pathological criteria for differentiation between grade II and III are difficult to apply and thus, their prognostic significance is of uncertain clinical utility [7]. Despite the fact that an array of histological or immunohistochemical markers have been proposed as prognostic factors [8–10], currently the prognostication of ependymoma is based only on clinical parameters i.e. age at diagnosis and extent of resection, with patients under 2 years of age and patients with residual tumor after surgery having a significantly poorer outcome [11, 12]. Thus knowledge about biological processes involved in ependymoma oncogenesis and novel biological prognostic and predictive markers are urgently needed [13]. Furthermore, different molecular changes associated with patient outcome have been detected by genetic studies [14–19] and different molecular subgroups with specific genomic alterations were identified. However, the application of these molecular subgroups in clinical practice is still limited [15, 19]. One major concern is the fact that most of the clinical studies on ependymoma included pediatric and adult cases and statistical analysis was often performed in these combined cohorts. However, it is now obvious that the course of the disease is different in pediatric and adult cases as it is reflected by a number of clinicopathological parameters. For instance in adult ependymomas supratentorial location is indicator of a worse prognosis while the limited number of studies with pediatric cases does not support this observation [9, 11]. It has also been demonstrated in a large cohort of pediatric and adult cases that potential histological prognosticators have a localization dependent role [20]. A recent study suggested that anaplastic morphology has a prognostic significance in pediatric infratentorial ependymomas [21].

The local invasive potential of cancer cells is critically determined by the cell-cell adhesion. In brain tumors the dysregulation of cell-cell adhesion proteins is a prerequisite for the invasion of the surrounding neural tissue [22, 23]. Claudins are pivotal components of the tight junction cell-cell adhesion complex. Currently 24 genes had been identified in the human genome that encodes claudins [24]. They regulate the paracellular barrier between the cells and thus the exchange of compounds through the intercellular junction. Claudin-1, claudin-2, claudin-5, claudin-7, claudin-11 and claudin-12 have so far been described in the central nervous system [25–27]. Claudin-5 and claudin-12 have been characterized as the only claudins of the brain endothelial cells suggesting a critical role in the formation of the blood brain barrier (BBB) [28]. Nevertheless, a number of claudins had been implicated in CNS tumorigenesis including claudin-1 and claudin-3 [29–32]. The potential clinical relevance of the expression of certain claudins (2–5, 7 and 10) expression was also studied in ependymomas and it is now evident that claudins can influence ependymoma cell growth [33].

There are certain regional differences described in the tight junction morphology and physiology of the ependymal epithelial cells lining the ventricular system including the choroid plexus. However, most of these studies have been performed in the rodent CNS [34–36]. A recent study indicated that there are important differences relevant to CNS tumor formation between mice and human ventricular zones [37]. However, these variations are not fully understood. Furthermore the BBB is not a tightly sealed system but there are specific regions that allow the direct contact of certain neurons with the cerebrospinal fluid (CSF) [38, 39]. Data on the molecular background of these spatial differences could contribute to the understanding of tumorigenesis and help to design more specific and thus efficient treatment options for pediatric ependymomas.

Accordingly, we analyzed the prognostic power of certain clinicopathological parameters and sought to characterize the expression of cell-cell adhesion proteins in normal ependyma as well as in pediatric ependymomas. Here we show that claudin-5 is highly expressed in the choroid plexus epithelium and in a subset of supratentorial ependymomas. Furthermore, we provide data about the influence of claudin-5 expression on overall survival in supratentorial pediatric ependymomas.

## Material and Methods

### Patients

The formalin fixed paraffin embedded (FFPE) histopathological samples from 54 pediatric patients with intracranial ependymomas were collected between 1997 and 2006 at the National Institute of Neurosurgery, Budapest, Hungary and between 1980 and 2000 at the Medical University of Vienna, Vienna, Austria. All histological samples were obtained from the initial surgery. No patients received radiation or chemotherapy therapy prior to the surgery. The average and median age of patients at the time of operation was 6.1 and 5 years, respectively (ranging from 8 months to 17 years). The average follow-up period was 6.8 years ranging from 2 weeks to 16.7 years.

### Ependyma Samples

Samples were collected from the routine autopsy of deceased newborns (35 to 40 gestation weeks). Subjects with central nervous system associated diseases were excluded from the study. The samples were collected in all cases from the right lateral, third and fourth ventricles and from the upper two segments of the cervical spinal cord. During careful anatomical opening of the ventricular system about 1 cm^3^ sized blocks were excised from the head of the caudate nucleus, choroid glomus attached to the crus of fornix, pes hippocampi, third ventricular surface of the thalamus and the medial eminence of the rhomboid fossa. Special care was taken to avoid damaging of the ependymal surface of the specimens. Choroid plexus tissue was also removed from the right lateral ventricle.

### Immunohistochemistry

Representative paraffin blocks - defined as those with the largest amount of viable and anaplastic tumor - for each tumor were selected. Immunohistochemistry was performed on the tumor tissues and the neonatal ependyma and choroid plexus. The immunohistochemical reactions were performed on 3 μm sections obtained from the FFPE blocks. After the deparaffinization steps, the slides were treated in a microwave oven in Target Retrieval Solution (S1699 from DAKO, Carpenteria, CA, USA) for 30 min for heat-induced epitope retrieval. The immunohistochemical reactions were performed in an automated Ventana ES Immunostainer System (Ventana Medical Systems Inc., Tucson, AZ, USA) with the solutions and steps according to the manufacturer. The following antibodies and dilutions were used: claudin-1 (1:80, Zymed, #18–7362), claudin-2 (1:20, Zymed, #18–7363), claudin-5 (1:120, Zymed, #18–7364), claudin-7 (1:100, Zymed, #34–9100), E-cadherin (1:500, Dako #M3612), N-cadherin (1:300, Abcam #12,221), occludin (1:250, Invitrogen, Carlsbad, CA, USA ) and vimentin (1:300, Dako #M0725). The slides were counterstained with Mayer’s hematoxylin (Zymed, South San Fransisco, CA, USA). Positive controls and negative control tissues (with the omission of the primary antibodies) were included in every run.

### Quantitative Real-Time PCR

Total RNA was isolated from ten macrodissected five-micron thick sections of FFPE blocks using RNeasy FFPE Kit according to the manufacturer’s instructions (QIAGEN, Hilden, Germany). The quantity and quality of the RNA was determined by NanoDrop. 400 nanogram of total RNA was used to perform reverse transcription using High Capacity RNA-to-cDNA Kit (Applied Biosystems Life Technologies, Carlsbad, CA, USA). Thirty nanograms of cDNA were used as a template for the real-time PCR using Power SYBR Green PCR Master Mix SYBRGreen on an ABI Prism 7000 Sequence Detection System. The following previously described claudin-5 and ABL primers were used: (5´ TTC CTG AAG TGG TGT CAC CTG AAC), reverse (5´ TGG CAG CTC TCA ATC TTC ACA G); forward (5´ ACG AGT CTG GTT GAT GCT GTG), reverse (5´ GGC GGA CTG TGG CTT TGG), respectively [40]. PCR reactions without cDNA samples were used as negative controls. Each reaction was performed in duplicate. The fluorescent data were converted into cycle threshold (CT) measurements, and the DDCT method was used to calculate expression relative to the internal control.

### Transmission Electronmicroscopy

Samples collected for ultrastructural studies were fixed in PBS containing 1 % paraformaldehyde and 1 % glutaraldehyde for 2 days at room temperature. Next, we dissected approximately 1 mm^3^ ependyma or choroid plexus containing tissue pieces that were transferred to calcium-cacodylate (Merck) for 3× 30 min and treated with 1 % OsO4 in cacodylate puffer for 2 h at 4 degree. Following washes with cacodylate solution (3 × 15 min) the samples were dehydrated in an ascending series of ethanol (30 %, 96 % and 100 % 2 × 30 min) and propylene oxide, propylene oxide-araldit, and embedded in Durcupan (ACM; Fluka, Buchs, Switzerland) at 56 degree for 2 days. A series of consecutive ultrathin sections (80 nm thick) were collected on Formvar-coated single-slot grids and contrasted by 6 % uranyl acetate in 50 % ethanol for 20 min and counterstained with lead citrate for 10 min. Electron micrographs were taken at a Hitachi (Yokohama, Japan) electron microscope.

### Statistics

The significance of the quantitative RT-PCR data was determined by Mann-Whitney test. The infratentorial and supratentorial group was compared by Mann-Whitney test and by χ^2^-test. Kaplan-Meier curves for overall survival were evaluated for the extent of resection, grade, tumor localization and claudin-5 expression in ependymoma cells for all patients in the study. Statistical significance (*P* < 0.05) was determined by log-rank test using GraphPad Prism 5.0 software (GraphPad Inc., San Diego, CA).

## Results

### Radical Resection and Supratentorial Localization Are Positive Prognosticators

**Fig. 1:**
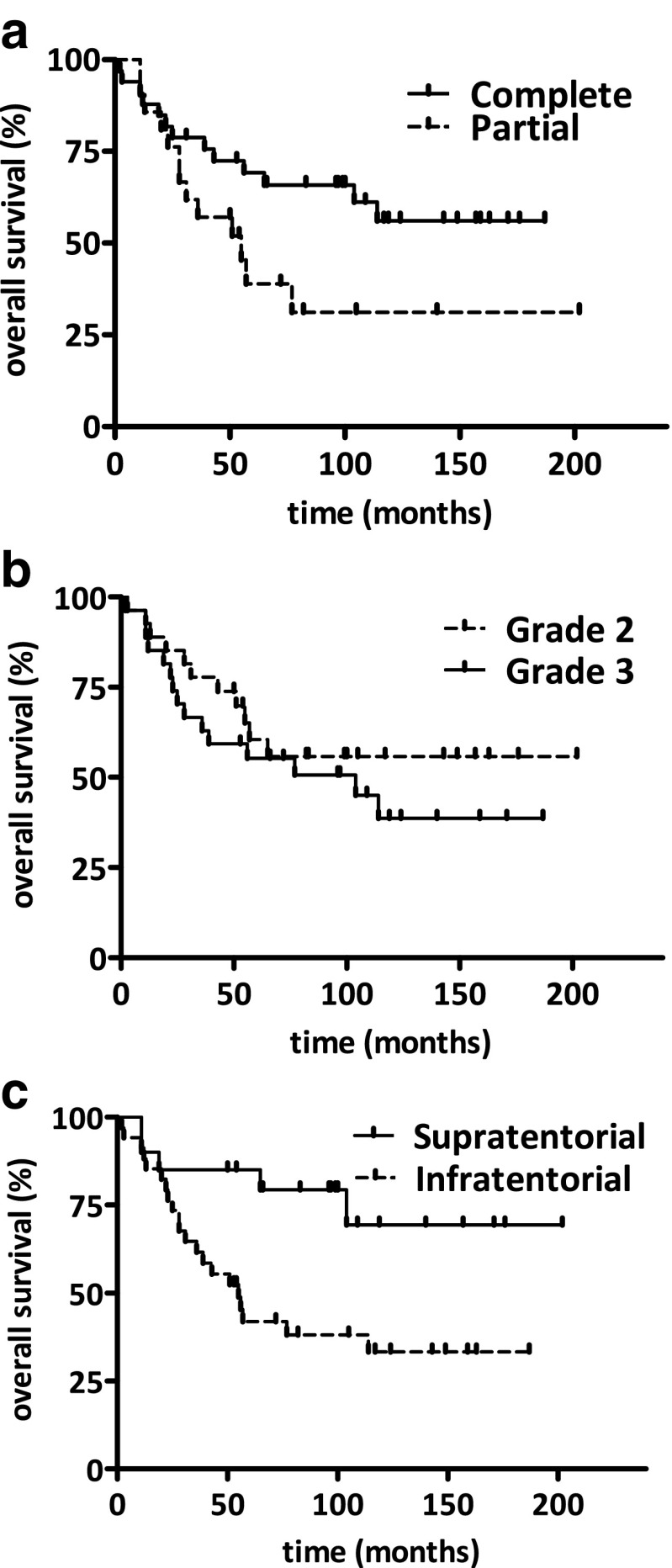
Kaplan-Meier analysis of overall survival in pediatric intracranial ependymomas. **a**, Complete surgical resection was found to be a positive prognostic marker as expected (*p* = 0.064) **b**, Histological grade did not predict the overall survival of patients (*p* = 0.35) **c**, Importantly, supratentorial localization is also a positive prognostic factor in our series (*p* = 0.013)

Out of the 54 patients, 33 (61.1 %) had undergone complete tumor resection based on the surgeons interpretation after completion of the procedure while in 21 cases (38.9 %) only partial resection had been achievable. In the Kaplan-Meyer there was a strong tendency for increased overall survival in the total resection group (Fig. 1a, p=0.064). Twenty seven (50 %) out of 54 tumors were categorized as grade 2 and 27 (50 %) as grade 3. There was no significant difference in overall survival between grade 2 and grade 3 ependymomas (Fig. 1b, p=0.35). There were 20 (37 %) supratentorial and 34 (63 %) infratentorial tumors in this series. The supratentorial group had significantly higher average age at diagnosis (*p* < 0.001) and a non-significantly higher proportion of total resection (*p* = 0.15) as presented in Table 1. There was no significant difference in the grade distribution between the supratentorial and infratentorial group (Table 1). Nevertheless, the supratentorial localization resulted in a significantly higher overall survival (Fig. 1c, p=0.013).

**Table 1:** Comparison of the infratentorial and supratentorial subgroup

Characteristics	Infratentorial (*N* = 34)		Supratentorial (*N* = 20)		*p*-value
Age (years)	3.9 ± 3.1		9.7 ± 4.5		<0.001
Grade					
II	17	50 %	10	50 %	1
III	17	50 %	10	50 %	
Resection					
Total	18	53 %	15	75 %	0.15
Subtotal	16	47 %	5	25 %	

### Claudin-5 Is differentially Expressed in Neonatal Ependymal Cells and Choroid Plexus Epithelia

**Fig. 2:**
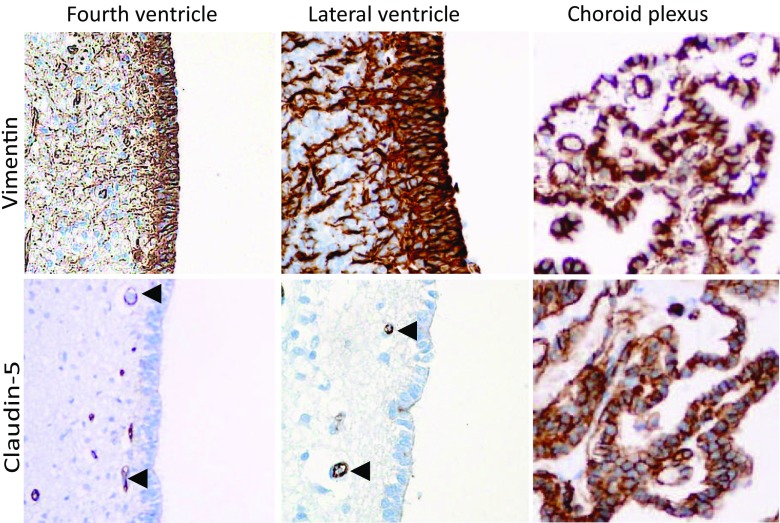
Claudin-5 expression in neonatal ependyma. We found no lateral claudin-5 staining in the ependymal cells of the ventricular system including the infratentorial fourth or supratentorial lateral ventricles. The endothelial cells of the subventricular capillaries are claudin-5 positive (indicated by *arrowheads*). In contrast, the choroid plexus epithelium showed intense staining along the lateral plasmamembrane as well. Vimentin staining identifies the ependymal cells as well as the brain blood vessels

**Fig. 3:**
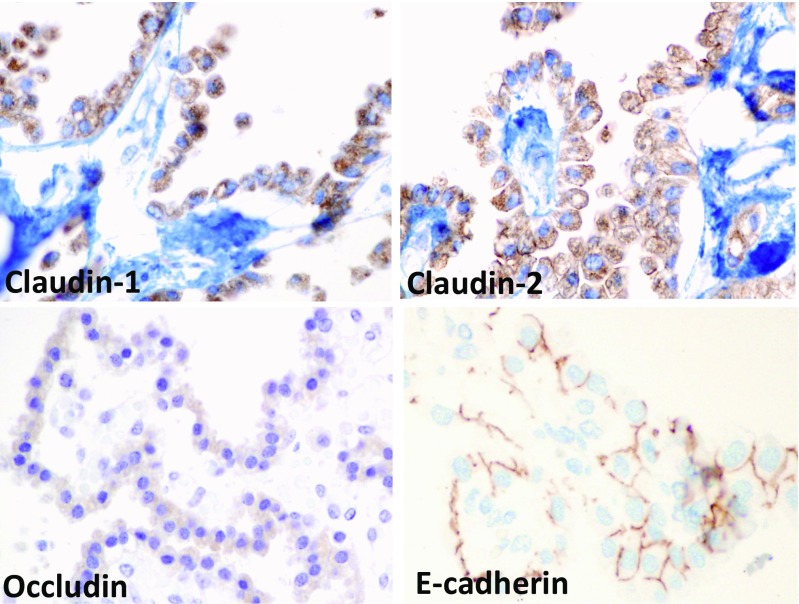
Cell-cell adhesion molecules in the neonatal choroid epithelium. Intense claudin-1 and claudin-2 labeling was observed in the plasma membrane and to some extent in the cytosol. Occludin staining was detected mostly in the apical region of the cells. E-cadherin clearly localized to the lateral plasma membrane

We hypothesized that regional variation in the expression of cell-cell adhesion molecules in the human ependyma may contribute to the differences in supratentorial and infratentorial ependymomas. We analyzed the expression of claudins (−1,-2,-5 and −7), E- and N-cadherin and occludin. We have collected central canal, ventricular wall and choroid plexus tissue samples from deceased neonates. The ependymal cell layer was identified by vimentin staining [41]. There was no lateral labeling of claudin-5 in any regions of the ventricular lining (Fig. 2). Interestingly choroid plexus epithelia displayed a very intense claudin-5 staining (Fig. 2). We could also demonstrate the expression of the other protein components of the tight junction complex including claudin-1, claudin-2 and occludin, but not claudin-7 in choroid plexus epithelia (Fig. 3).

### Distinct Ultrastructural Composition of Cell-Cell Junction in Neonatal Ependyma and Choroid Plexus Epithelium

**Fig. 4:**
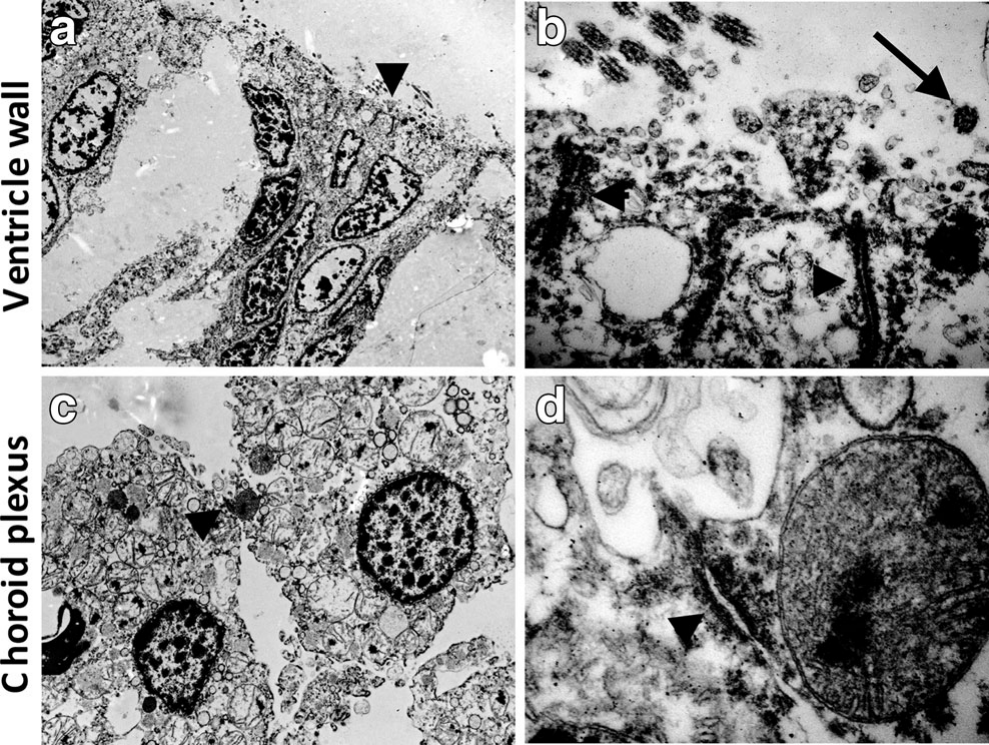
Ultrastructural differences in the cell-cell junction in the neonatal ependymal cell layer and choroid epithelium. Choroid plexus epithelium and ventricular ependymal cells possess distinct cell-cell adhesion structures in the lateral plasma membrane as indicated by arrowheads. Note the lack of tight junctions in the ventricular ependymal cells whereas they display adherent junctions. Both cell types carry microvilli on the apical surface. However, in contrast to ventricular ependymal cells, the choroid plexus epithelium lacks cilia (indicated by *arrow*)

Next we sought to analyze whether regional differences in the expression of cell-cell junction molecules in ependyma and choroid plexus lead to altered cell-cell junction morphology and function. Choroid plexus epithelium and ventricular ependymal cells possess distinct cell-cell junction complexes in the apico-lateral plasma membrane as depicted in Fig. 4. The ventricular ependymal cells lack tight junctions but display rather large adherent junctions. Both cell types display microvilli on the apical surface. As expected, only the ventricular ependymal cells carry cilia while the choroid plexus epithelium lacks these organelles.

### Claudin-5 Is Expressed in a Subset of Supratentorial Pediatric Ependymomas

**Fig. 5:**
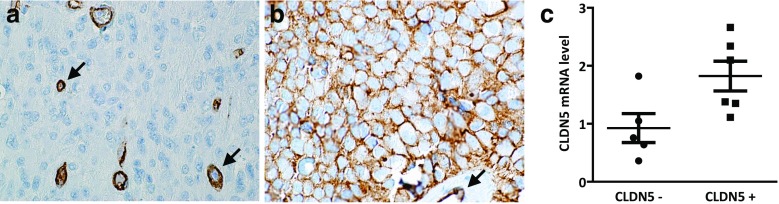
Claudin-5 expression in pediatric intracranial ependymomas. **a**, In 34 infratentorial ependymomas only the brain endothelial cells expressed claudin-5. **b**, In contrast, in 9 out of 20 supratentorial cases the ependymal cells displayed plasma membrane localized claudin-5 expression. *Arrows* indicate the blood vessels. **c**, A significant increase in claudin-5 expression was found at the transcription level by qRT-PCR (*p* = 0.03). Due to the high level of endothelial cell expression even claudin-5 negative ependymoma samples contained relatively high amount of CLDN-5 mRNA

Claudin-5 is displayed in the endothelial cells of the tumor capillaries of the ependymoma tissue (Fig. 5a and b, arrowheads). Importantly, in 9 (45 %) of the 20 supratentorial cases the ependymoma tumor cells expressed claudin-5 with appropriate plasma membrane localization (Fig. 5b). Furthermore, we could not detect expression of claudin-1 and −2, occludin or E- and N-cadherin in these cases suggesting that claudin-5 expression delineates a specific subset of ependymoma cases. Importantly, none of the infratentorial ependymomas showed claudin-5 expression (Fig. 5a). Additionally, we have also analyzed 9 spinal ependymomas and found no claudin-5 expression in the tumor cells (data not shown). We next isolated total RNA from macrodissected FFPE sections of 5 claudin-5 negative and 6 claudin-5 positive cases. The average expression level of claudin-5 determined by quantitative RT-PCR was significantly increased in the positive cases (Fig. 5c.). Importantly, other major components of the cell-cell junction complex (claudin-1, −2, and −7, occludin, E- and N-cadherin) did not show this region specific expression (data not shown).

### Claudin-5 Expressing Ependymomas Tend to Display a longer Overall Survival

**Fig. 6:**
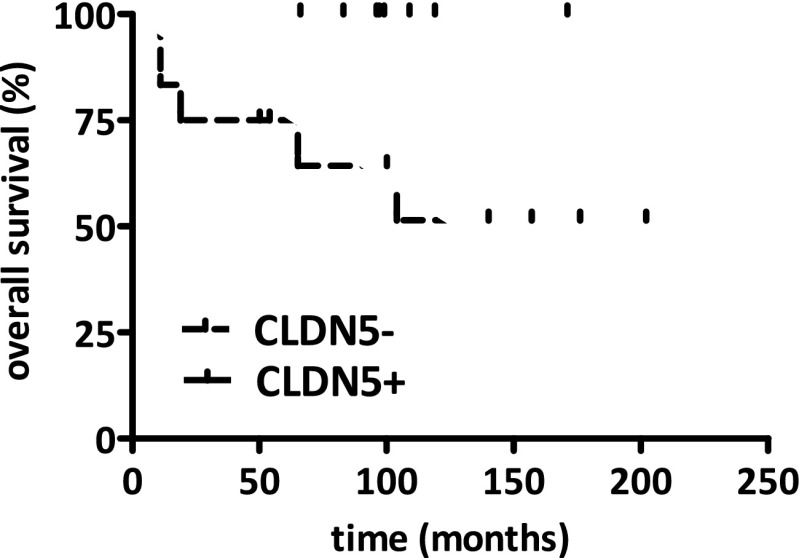
Kaplan-Meier analysis of overall survival and claudin-5 expression in supratentorial pediatric ependymomas. The claudin-5 positive supratentorial group displayed an increased overall survival when compared to the claudin-5 negative supratentorial cases (*p* = 0.048)

Since cell-cell junction complexes are critical regulators of tissue cohesion and influence the invasive potential of cells we investigated whether localization and claudin-5 expression can influence the progression of the disease as characterized by overall survival. Interestingly, the claudin-5 negative supratentorial cases showed a decreased overall survival in the Kaplan-Meier survival analysis when compared to claudin-5 positive tumors (Fig. 6).

## Discussion

The recent revolution in molecular profiling of ependymomas has led to the identification of new clinically relevant subsets of tumors [15, 19, 42]. Gene expression microarray studies on ependymoma have implicated radial glial cells as the cell of origin for ependymomas [43]. Furthermore, a distinct molecular profile has been established for ependymomas with different tumor grades and localization [42]. Also, distinct pattern of hypermethylation was found in spinal, posterior fossa and supratentorial ependymomas [44]. A recent comparative genome wide methylation and gene expression microarray study identified novel differentially regulated genes in pediatric ependymomas [45]. The gain of 1q was suggested to be associated with survival in intracranial ependymoma, however, not all subsequent studies could confirm the prognostic power of this genomic alteration [16, 46]. Altogether, none of these studies yielded new molecular prognostic markers for pediatric ependymomas so far.

Here we show that claudin-5 is expressed by choroid plexus epithelial cells but not by ventricular ependymal cells. So far only claudin-1, −2, and −11 had been described in choroid plexus epithelial cells as part of the blood-CSF barrier and claudin-3 and claudin-5 in brain endothelial cells as a component of the blood-brain barrier [27, 36]. The demonstrated ultrastructural and immunohistochemical differences between the choroid plexus epithelia and the ventricular ependymal cells are in line with functional observations. Namely, the ventricular ependymal cells do not form a tight barrier between the CSF and the nervous system interstitia, while the epithelial cells of the choroid plexus form a highly regulated barrier between the CSF and the blood vessels and thus are major contributors to the blood-CSF barrier [34, 47].

In our study we confirmed that claudin-5 is expressed by a subset of supratentorial ependymomas [33] and provide evidence that it is not expressed in infratentorial tumors. In line with previous findings, ependymoma retains some of the region specific differences that can be found in the radial glial cell populations [43]. Interestingly, a previous gene expression signature had been established in a microarray study that distinguished spinal, posterior fossa and supratentorial ependymal tumors and found claudin-5 specifically overexpressed in supratentorial cases (supplemental data in [43]). The presence of the tight junction component claudin-5 might contribute to increased cell-cell adhesion between the claudin-5 expressing ependymoma tumor cells and thus interfere with the invasive potential of ependymoma cells.

There is emerging evidence that the innate regional molecular differences in the progenitor cell populations of the central nervous system play a pivotal role in the development of pediatric brain tumors [48–50]. Our observation that a subset of supratentorial ependymomas expresses claudin-5 and displays a different biological behavior may suggest that these ependymomas arise from a distinct progenitor population of the human ventricular system.

Furthermore, recent studies have identified stem-cell like tumor cells in glioblastoma tumors that formed vessel-like structures with connection to the circulation. The expression of a brain endothelial cell tight junction component by ependymoma cells may suggest the existence of this kind of molecular vascular mimicry in pediatric ependymomas.

In our series of tumors the extent of resection and the localization was found to be a prognostic factor. These observations are in line with previous studies that analyzed the prognostic significance of clinicopathological variables in pediatric ependymoma cases [1, 10, 21]. Similar to other studies, the grade was not predicting the overall survival in our patient population [51]. However, here we demonstrate that claudin-5 expression by ependymoma cells is a potential predictor of overall survival.

Altogether, our data show that claudin-5 expression in pediatric intracranial ependymoma is a potential prognostic factor and should be further evaluated as a promising histopathological biomarker. Furthermore, in prospective clinical trials with pediatric ependymomas claudin-5 expression should also be registered to evaluate whether this subset of ependymomas displays a different response for adjuvant treatments.

## References

[1] Bergeron C, Philip T (2004). Childhood cancer. Epidemiologic, diagnostic, and therapeutic aspects. La Revue du praticien.

[2] Maksoud YA, Hahn YS, Engelhard HH (2002). Intracranial ependymoma. Neurosurg Focus.

[3] Gelabert Gonzalez M, Garcia Allut A, Fernandez Villa JM, Gonzalez Garcia J, Martinez Rumbo R (2001). Intracranial ependymomas. Rev Neurol.

[4] Yang CP, Hung IJ, Jaing TH, Shih LY, Chang WH (2005). Cancer in infants: a review of 82 cases. Pediatr Hematol Oncol.

[5] Louis DN, Ohgaki H, Wiestler OD, Cavenee WK, Burger PC, Jouvet A, Scheithauer BW, Kleihues P (2007). The 2007 WHO classification of tumours of the central nervous system. Acta Neuropathol.

[6] Kilday JP, Rahman R, Dyer S, Ridley L, Lowe J, Coyle B, Grundy R (2009). Pediatric ependymoma: biological perspectives. Mol Cancer Res.

[7] Godfraind C (2009). Classification and controversies in pathology of ependymomas. Childs Nerv Syst.

[8] Preusser M, Heinzl H, Gelpi E, Hoftberger R, Fischer I, Pipp I, Milenkovic I, Wohrer A, Popovici F, Wolfsberger S, Hainfellner JA (2008). Ki67 index in intracranial ependymoma: a promising histopathological candidate biomarker. Histopathology.

[9] Milde T, Hielscher T, Witt H, Kool M, Mack SC, Deubzer HE, Oehme I, Lodrini M, Benner A, Taylor MD, von Deimling A, Kulozik AE, Pfister SM, Witt O, Korshunov A (2012) Nestin expression identifies ependymoma patients with poor outcome. Brain Pathol. doi:10.1111/j.1750-3639.2012.00600.x 10.1111/j.1750-3639.2012.00600.x PMC8057643 22568867

[10] Ridley L, Rahman R, Brundler MA, Ellison D, Lowe J, Robson K, Prebble E, Luckett I, Gilbertson RJ, Parkes S, Rand V, Coyle B, Grundy RG (2008). Multifactorial analysis of predictors of outcome in pediatric intracranial ependymoma. Neuro-Oncology.

[11] Figarella-Branger D, Civatte M, Bouvier-Labit C, Gouvernet J, Gambarelli D, Gentet JC, Lena G, Choux M, Pellissier JF (2000). Prognostic factors in intracranial ependymomas in children. J Neurosurg.

[12] Jaing TH, Wang HS, Tsay PK, Tseng CK, Jung SM, Lin KL, Lui TN (2004). Multivariate analysis of clinical prognostic factors in children with intracranial ependymomas. J Neuro-Oncol.

[13] Kuncova K, Janda A, Kasal P, Zamecnik J (2009). Immunohistochemical prognostic markers in intracranial ependymomas: systematic review and meta-analysis. Pathol Oncol Res.

[14] Carter M, Nicholson J, Ross F, Crolla J, Allibone R, Balaji V, Perry R, Walker D, Gilbertson R, Ellison DW (2002). Genetic abnormalities detected in ependymomas by comparative genomic hybridisation. Br J Cancer.

[15] Korshunov A, Witt H, Hielscher T, Benner A, Remke M, Ryzhova M, Milde T, Bender S, Wittmann A, Schottler A, Kulozik AE, Witt O, von Deimling A, Lichter P, Pfister S (2010). Molecular staging of intracranial ependymoma in children and adults. J Clin Oncol.

[16] Mendrzyk F, Korshunov A, Benner A, Toedt G, Pfister S, Radlwimmer B, Lichter P (2006) Identification of gains on 1q and epidermal growth factor receptor overexpression as independent prognostic markers in intracranial ependymoma. Clin Cancer Res 12 (7 Pt 1):2070–2079. doi:10.1158/1078-0432.CCR-05-2363 10.1158/1078-0432.CCR-05-2363 16609018

[17] Monoranu CM, Huang B, Zangen IL, Rutkowski S, Vince GH, Gerber NU, Puppe B, Roggendorf W (2008). Correlation between 6q25.3 deletion status and survival in pediatric intracranial ependymomas. Cancer Genet Cytogenet.

[18] Puget S, Grill J, Valent A, Bieche I, Dantas-Barbosa C, Kauffmann A, Dessen P, Lacroix L, Geoerger B, Job B, Dirven C, Varlet P, Peyre M, Dirks PB, Sainte-Rose C, Vassal G (2009). Candidate genes on chromosome 9q33–34 involved in the progression of childhood ependymomas. J Clin Oncol.

[19] Witt H, Mack SC, Ryzhova M, Bender S, Sill M, Isserlin R, Benner A, Hielscher T, Milde T, Remke M, Jones DT, Northcott PA, Garzia L, Bertrand KC, Wittmann A, Yao Y, Roberts SS, Massimi L, Van Meter T, Weiss WA, Gupta N, Grajkowska W, Lach B, Cho YJ, von Deimling A, Kulozik AE, Witt O, Bader GD, Hawkins CE, Tabori U, Guha A, Rutka JT, Lichter P, Korshunov A, Taylor MD, Pfister SM (2011). Delineation of two clinically and molecularly distinct subgroups of posterior fossa ependymoma. Cancer Cell.

[20] Raghunathan A, Wani K, Armstrong TS, Vera-Bolanos E, Fouladi M, Gilbertson R, Gajjar A, Goldman S, Lehman NL, Metellus P, Mikkelsen T, Necesito-Reyes MJ, Omuro A, Packer RJ, Partap S, Pollack IF, Prados MD, Robins HI, Soffietti R, Wu J, Miller CR, Gilbert MR, Aldape KD, Collaborative Ependymoma Research N (2013). Histological predictors of outcome in ependymoma are dependent on anatomic site within the central nervous system. Brain Pathol.

[21] Phi JH, Wang KC, Park SH, Kim IH, Kim IO, Park KD, Ahn HS, Lee JY, Son YJ, Kim SK (2012). Pediatric infratentorial ependymoma: prognostic significance of anaplastic histology. J Neuro-Oncol.

[22] Maret D, Gruzglin E, Sadr MS, Siu V, Shan W, Koch AW, Seidah NG, Del Maestro RF, Colman DR (2010). Surface expression of precursor N-cadherin promotes tumor cell invasion. Neoplasia.

[23] Lewis-Tuffin LJ, Rodriguez F, Giannini C, Scheithauer B, Necela BM, Sarkaria JN, Anastasiadis PZ (2010). Misregulated E-cadherin expression associated with an aggressive brain tumor phenotype. PLoS One.

[24] Krause G, Winkler L, Mueller SL, Haseloff RF, Piontek J, Blasig IE (2008). Structure and function of claudins. Biochim Biophys Acta.

[25] Liebner S, Fischmann A, Rascher G, Duffner F, Grote EH, Kalbacher H, Wolburg H (2000). Claudin-1 and claudin-5 expression and tight junction morphology are altered in blood vessels of human glioblastoma multiforme. Acta Neuropathol.

[26] Takei H, Bhattacharjee MB, Rivera A, Dancer Y, Powell SZ (2007). New immunohistochemical markers in the evaluation of central nervous system tumors: a review of 7 selected adult and pediatric brain tumors. Arch Pathol Lab Med.

[27] Wolburg H, Wolburg-Buchholz K, Kraus J, Rascher-Eggstein G, Liebner S, Hamm S, Duffner F, Grote EH, Risau W, Engelhardt B (2003). Localization of claudin-3 in tight junctions of the blood-brain barrier is selectively lost during experimental autoimmune encephalomyelitis and human glioblastoma multiforme. Acta Neuropathol.

[28] Szmydynger-Chodobska J, Pascale CL, Pfeffer AN, Coulter C, Chodobski A (2007) Expression of junctional proteins in choroid plexus epithelial cell lines: a comparative study. Cerebrospinal Fluid Res 4:11 10.1186/1743-8454-4-11 PMC2241822 18162136

[29] Schulzke JD, Fromm M (2009) Tight junctions: molecular structure meets function. Annals of the New York Academy of Sciences 1165:1–6 10.1111/j.1749-6632.2009.04925.x 19538280

[30] Fanning AS, Mitic LL, Anderson JM (1999). Transmembrane proteins in the tight junction barrier. J Am Soc Nephrol.

[31] Forster C (2008). Tight junctions and the modulation of barrier function in disease. Histochem Cell Biol.

[32] Gonzalez-Mariscal L, Betanzos A, Nava P, Jaramillo BE (2003). Tight junction proteins. Prog Biophys Mol Biol.

[33] Nordfors K, Haapasalo J, Sallinen PK, Haapasalo H, Soini Y (2013). Expression of claudins relates to tumour aggressivity, location and recurrence in ependymomas. Histol Histopathol.

[34] Masseguin C, Mani-Ponset L, Herbute S, Tixier-Vidal A, Gabrion J (2001). Persistence of tight junctions and changes in apical structures and protein expression in choroid plexus epithelium of rats after short-term head-down tilt. J Neurocytol.

[35] Mathew TC (2008). Regional analysis of the ependyma of the third ventricle of rat by light and electron microscopy. Anat Histol Embryol.

[36] Wolburg H, Wolburg-Buchholz K, Liebner S, Engelhardt B (2001). Claudin-1, claudin-2 and claudin-11 are present in tight junctions of choroid plexus epithelium of the mouse. Neurosci Lett.

[37] Dahiya S, Lee d Y, Gutmann DH (2011). Comparative characterization of the human and mouse third ventricle germinal zones. J Neuropathol Exp Neurol.

[38] Vigh B, Manzano e Silva MJ, Frank CL, Vincze C, Czirok SJ, Szabo A, Lukats A, Szel A (2004). The system of cerebrospinal fluid-contacting neurons. Its supposed role in the nonsynaptic signal transmission of the brain. Histol Histopathol.

[39] Vigh B, Vigh-Teichmann I (1998). Actual problems of the cerebrospinal fluid-contacting neurons. Microsc Res Tech.

[40] Patonai A, Erdelyi-Belle B, Korompay A, Somoracz A, Straub BK, Schirmacher P, Kovalszky I, Lotz G, Kiss A, Schaff Z (2011). Claudins and tricellulin in fibrolamellar hepatocellular carcinoma. Virchows Arch.

[41] Schnitzer J, Franke WW, Schachner M (1981). Immunocytochemical demonstration of vimentin in astrocytes and ependymal cells of developing and adult mouse nervous system. J Cell Biol.

[42] Palm T, Figarella-Branger D, Chapon F, Lacroix C, Gray F, Scaravilli F, Ellison DW, Salmon I, Vikkula M, Godfraind C (2009). Expression profiling of ependymomas unravels localization and tumor grade-specific tumorigenesis. Cancer.

[43] Taylor MD, Poppleton H, Fuller C, Su X, Liu Y, Jensen P, Magdaleno S, Dalton J, Calabrese C, Board J, Macdonald T, Rutka J, Guha A, Gajjar A, Curran T, Gilbertson RJ (2005). Radial glia cells are candidate stem cells of ependymoma. Cancer Cell.

[44] Rogers HA, Kilday JP, Mayne C, Ward J, Adamowicz-Brice M, Schwalbe EC, Clifford SC, Coyle B, Grundy RG (2012). Supratentorial and spinal pediatric ependymomas display a hypermethylated phenotype which includes the loss of tumor suppressor genes involved in the control of cell growth and death. Acta Neuropathol.

[45] Pérez-Ramírez M, Hernández-Jiménez AJ, Guerrero-Guerrero A, Benadón-Darszon E, Pérezpeña-Díazconti M, Siordia-Reyes AG, García-Méndez A, de León FC-P, Salamanca-Gómez FA, García-Hernández N (2016) Genomics and epigenetics: A study of ependymomas in pediatric patients. Clin Neurol Neurosurg 144:53–58. doi:10.1016/j.clineuro.2016.02.041 10.1016/j.clineuro.2016.02.041 26971296

[46] Rajeshwari M, Sharma MC, Kakkar A, Nambirajan A, Suri V, Sarkar C, Singh M, Saran RK, Gupta RK (2016). Evaluation of chromosome 1q gain in intracranial ependymomas. J Neuro-Oncol.

[47] Lippoldt A, Liebner S, Andbjer B, Kalbacher H, Wolburg H, Haller H, Fuxe K (2000). Organization of choroid plexus epithelial and endothelial cell tight junctions and regulation of claudin-1, −2 and −5 expression by protein kinase C. Neuroreport.

[48] Lee d Y, Gianino SM, Gutmann DH (2012). Innate neural stem cell heterogeneity determines the patterning of glioma formation in children. Cancer Cell.

[49] Sharma MK, Mansur DB, Reifenberger G, Perry A, Leonard JR, Aldape KD, Albin MG, Emnett RJ, Loeser S, Watson MA, Nagarajan R, Gutmann DH (2007). Distinct genetic signatures among pilocytic astrocytomas relate to their brain region origin. Cancer Res.

[50] Gibson P, Tong Y, Robinson G, Thompson MC, Currle DS, Eden C, Kranenburg TA, Hogg T, Poppleton H, Martin J, Finkelstein D, Pounds S, Weiss A, Patay Z, Scoggins M, Ogg R, Pei Y, Yang ZJ, Brun S, Lee Y, Zindy F, Lindsey JC, Taketo MM, Boop FA, Sanford RA, Gajjar A, Clifford SC, Roussel MF, McKinnon PJ, Gutmann DH, Ellison DW, Wechsler-Reya R, Gilbertson RJ (2010). Subtypes of medulloblastoma have distinct developmental origins. Nature.

[51] Ellison DW, Kocak M, Figarella-Branger D, Felice G, Catherine G, Pietsch T, Frappaz D, Massimino M, Grill J, Boyett JM, Grundy RG (2011) Histopathological grading of pediatric ependymoma: reproducibility and clinical relevance in European trial cohorts. J Negat Results Biomed 10(7). doi:10.1186/1477-5751-10-7 10.1186/1477-5751-10-7 PMC3117833 21627842

